# Bidirectional Associations Between Primary Caregivers’ and Adolescents’ Depressive and Anxiety Symptoms: An Intergenerational Actor–Partner Interdependence Model Analysis

**DOI:** 10.1155/da/6987736

**Published:** 2026-05-30

**Authors:** Hao Hou, Dan Luo, Shuzhen Zhu, Si Chen Zhou, Shu Yan, Yu Lei Jiang, Xiao Qin Wang, Qian Liu, Huijing Zou, Bing Xiang Yang

**Affiliations:** ^1^ Department of Psychiatry, Renmin Hospital of Wuhan University, Wuhan, China, rmhospital.com; ^2^ Center for Wise Information Technology of Mental Health Nursing Research, School of Nursing, Wuhan University, Wuhan, Hubei, China, whu.edu.cn; ^3^ Research Center for Lifespan Health, Wuhan University, Wuhan, China, whu.edu.cn; ^4^ Institute of Chronic and Non-communicable Disease Control and Prevention, Hubei Provincial Center for Disease Control and Prevention, Wuhan, Hubei, China, cdc.gov; ^5^ Office of Psychosocial Services, Wuhan Mental Health Center, Wuhan, Hubei Province, China; ^6^ Office of Psychosocial Services, Wuhan Hospital for Psychotherapy, Wuhan, Hubei Province, China

**Keywords:** adolescents, anxiety, caregivers, depression, intergenerational transmission

## Abstract

**Background:**

Extensive research has focused on the top‐down intergenerational transmission of psychopathological symptoms (ITP). However, little is known about the bidirectional and transactional dynamics of ITP between primary caregivers and their adolescent offspring in community‐based samples. In particular, the moderating roles of caregiver and adolescent gender in these intergenerational associations remain underexplored.

**Methods:**

Between 2021 and 2022, a total of 1414 primary caregiver–adolescent dyads completed two waves of surveys assessing sociodemographic characteristics, as well as depressive and anxiety symptoms. The actor–partner interdependence model (APIM) was applied to examine the bidirectional transactional patterns of psychopathological symptoms within these dyads. Multiple‐group comparison tests were conducted to evaluate the moderating effects of caregiver and adolescent gender on these associations.

**Results:**

Adolescents’ depressive and anxiety symptoms significantly predicted subsequent depressive and anxiety symptoms in their primary caregivers. In contrast, only caregivers’ anxiety symptoms were found to predict subsequent adolescents’ depressive and anxiety symptoms. Multiple‐group comparison tests indicated no significant moderating effects of caregiver or adolescent gender on the ITP.

**Conclusion:**

These findings suggest that depressive and anxiety symptoms may be mutually reinforced between adolescents and primary caregivers over time. A family‐based approach to early identification and prevention could be a promising direction for interrupting escalating cycles of negative emotions across generations.

## 1. Introduction

Adolescents are particularly vulnerable to depressive and anxiety symptoms due to rapid physiological, psychological, and social changes during this developmental period [1]. Research has indicated that ~25.2% of adolescents suffer from depressive symptoms and 20.5% from anxiety symptoms [2]. Notably, such symptoms often cluster within families, where adolescents and caregivers mutually influence each other’s emotional well‐being [3]. These dynamics may be attributed to the intergenerational transmission of psychopathological symptoms (ITP), a process by which mental health symptoms are transmitted across generations through genetic, environmental, and psychological mechanisms [4].

### 1.1. Bidirectional Intergenerational Transmission of Depressive and Anxiety Symptoms

Parental traits shape the environments in which children are raised, eliciting predictable cognitive, emotional, and behavioral responses from the child [5]. In turn, these child responses can serve as catalysts for changes in parental behaviors and characteristics [5].

Numerous studies have examined the impact of caregivers’ emotional symptoms on their offspring—demonstrating that both paternal and maternal depression can contribute to increased depressive and anxiety symptoms in children from infancy through adolescence [6–8]. However, most existing research often relies on unidirectional frameworks and fails to consider how adolescents’ symptoms may influence caregivers over time [4, 9, 10]. The limited evidence focusing on the bidirectional associations in ITP remains inconsistent. For instance, Johnco et al. [11] found that parental anxiety was associated with increases in child anxiety and depression, but there was no bidirectional association from child psychopathology to parental anxiety, whereas Hastings et al. [12] reported a bidirectional association between maternal anxiety and subsequent youth anxiety and depression.

Therefore, the bidirectional nature of ITP remains largely unclear. Additionally, previous studies have several limitations. First, many studies were conducted with relatively small sample sizes [11, 12], which may compromise the stability and generalizability of the findings. Second, these studies were often carried out in varying sociocultural contexts [11, 12]. Given that ITP may be closely linked to culturally specific parenting styles [11, 13], existing findings may lack cross‐cultural applicability.

### 1.2. Moderating Roles of Caregiver and Adolescent Gender in ITP

Research suggested that the ITP may vary depending on the gender of both caregivers and adolescents. In traditional cultures, mothers are often primary emotional caregivers, while fathers are more involved in discipline or goal‐oriented parenting [14]. These role distinctions may contribute to different ITP patterns. For example, Hastings et al. [12] reported no significant associations between paternal and youth psychopathology. However, others found that depressive symptoms in fathers were also associated with offspring internalizing symptoms [6, 15].

Beyond caregiver roles, adolescents’ gender may also play as a moderator in ITP. The gender intensification hypothesis posits that girls are more susceptible to internalizing symptoms due to heightened social expectations during adolescence [16]. For example, both Hastings et al. [12] and Felton et al. [7] found that maternal depression predicted depressive symptoms in daughters but not in sons. However, some studies have reported contrasting findings that parental depression was associated with an increased risk of depression in boys but not in girls [17]. In contrast, Currier et al. [18] argued that there were no significant gender differences in the ITP.

Thus, the moderating role of adolescent and caregiver gender in ITP remains unclear. Yet, few studies have systematically examined whether caregiver and adolescent gender can moderate ITP, particularly within non‐Western family contexts. Addressing this gap is crucial for developing interventions that are sensitive to both gender and cultural differences.

### 1.3. Actor–Partner Interdependence Model (APIM)

A methodological limitation in previous research lies in the assumption of independence in statistical models. Family members function as interdependent systems, where parental and adolescent depressive and anxiety symptoms are mutually influenced. However, many studies have used regression models that assume independence among observations, which may lead to biased estimates [11, 19]. The APIM offers a more appropriate framework by simultaneously estimating actor effects (how an individual’s symptoms affect their own outcomes) and partner effects (how they affect the outcomes of others in the dyad) [20, 21]. This approach has been widely applied in research on spousal, parent–child, and patient–caregiver dyads [22–24], but its use in ITP research remains limited.

## 2. Current Study

This study addressed several limitations of previous research, including small sample sizes and methodological constraints, by systematically examining the bidirectional associations of psychopathological symptoms and the moderating roles of both caregiver and adolescent gender within the Chinese cultural context. Understanding these reciprocal pathways is critical for identifying key intervention targets to disrupt negative emotional cycles within families.

We hypothesized that

1. depressive and anxiety symptoms would be bidirectionally associated between adolescents and their primary caregivers and
2. caregiver and adolescent gender would moderate these transmission effects.

## 3. Methods

### 3.1. Study Setting and Participants

This study is based on a longitudinal study titled the *Students’ Mental Health Network* (SMHN) carried out in Wuhan, China. The aim of SMHN is to integrate the efforts of parents, schools, and hospitals to promote the mental health of students through a regular assessment of their mental health. A baseline survey (T1) was completed from September to October 2021. Eight schools (four junior high schools and four senior high schools) were included in the SMHN. A cluster sampling method was employed to recruit students and one of their primary caregivers (limited to one parent per adolescent). All subjects were informed of the right to withdraw at any time, and informed consent was obtained electronically. To ensure the confidentiality of data, it was collected using a proprietary electronic platform of the SMHN. Adolescents were taken to a computer room for data collection, while caregivers were asked to complete data collection using their cell phones during a fixed period. The survey platform was programed to prompt participants to complete any missing responses and automatically redirect them as needed, thereby ensuring complete data collection. As a result, there were no missing values in this study.

Using a unique identifier in the questionnaire, students could be individually matched with their primary caregiver. From September to October 2022 (1 year after baseline), the SMHN survey was repeated in the same way (T2).

Exclusion criteria were: (1) unreliable answers (logic error); (2) questionnaires of caregivers and students cannot be matched; (3) caregivers were not the father or mother; (4) student or caregivers lost to follow‐up; and (5) caregiver at baseline and follow‐up does not match. A total of 1414 pairs of parent–child pairs were included in the study.

The Clinical Research Ethics Committee of the Wuhan Mental Health Center reviewed and approved the study protocol (KY2021.11.01).

### 3.2. Instruments

#### 3.2.1. Sociodemographic Questionnaire

A structured questionnaire was researcher‐designed to obtain sociodemographic data on adolescents and their caregivers. The adolescents’ questionnaire included gender (male/female), grades (junior high school/senior high school), only child (yes/no), and the role of caregivers (father/mother). The caregivers’ questionnaire included parental education level (junior high school or below, senior high school or associate degree, and bachelor’s degree or higher) and annual income of family in yuan [7.2 = US$] (<80,000, 80,000–150,000. 150,000−300,000, >300,000).

#### 3.2.2. Patient Health Questionnaire‐9 (PHQ‐9)

The PHQ‐9 was used to assess the depressive symptom level of the caregiver–adolescent dyads. The Chinese version of PHQ‐9 was used in the current study and has shown good reliability and validity [25, 26]. The scale includes nine items assessing the main symptoms of depression (anhedonia, sad mood, sleep, fatigue, appetite, guilt, concentration, motor, and suicidal ideation). Each item was rated on a standard 4‐point Likert rating scale from 0 (not at all) to 3 (nearly every day). The total score is the sum of item scores, with a higher total score indicating more severe depressive symptoms. Cronbach’s α of the adolescents and caregivers of the PHQ‐9 at baseline were 0.885 and 0.874 and 0.892 and 0.874 at follow‐up, respectively.

#### 3.2.3. Generalized Anxiety Disorder Scale (GAD‐7)

Anxiety symptoms of caregiver‐adolescent dyads were assessed using the Chinese version of the GAD‐7 [27, 28], which has proven good reliability and validity [27]. The GAD‐7 assesses seven main symptoms of anxiety (nervousness, controlled worry, worry a lot, relaxation, restlessness, irritability, and being afraid). It uses a 4‐point Likert scale from 0 (not at all) to 3 (nearly every day). The total score is the sum of item scores, with a higher score indicating a higher level of anxiety. Cronbach’s α of the students’ and caregivers’ GAD‐7 were 0.916 and 0.933 at baseline and 0.925 and 0.915 at follow‐up, respectively.

### 3.3. Analysis Strategies

Participants were divided into two groups by the role of the primary caregiver (father as primary caregiver and mother as primary caregiver). Descriptive analysis was used to summarize the sociodemographic characteristics and mental health conditions. The Mann–Whitney *U* test and chi‐square test were used to examine the difference in sociodemographic characteristics and mental health conditions between two groups. The Wilcoxon signed‐rank test was used in a longitudinal comparison within each group.

APIM was employed to examine the reciprocal associations between caregivers’ and adolescents’ depressive and anxiety symptoms from T1 to T2. All models included autoregressive paths representing the stability of psychological symptoms over time (actor effects) as well as cross‐lagged predictive paths between caregivers’ and adolescents’ psychological symptoms from T1 to T2 (partner effects). The bootstrapping method generated 5000 randomized samples, and the full information maximum likelihood estimation method was used to estimate the APIM models.

The researchers constructed four APIM models to examine both single‐disorder (adolescents’ depressive symptoms with caregivers’ depressive symptoms and adolescents’ anxiety symptoms with caregivers’ anxiety symptoms) and cross‐disorder (adolescents’ depressive symptoms with caregivers’ anxiety symptoms and adolescents’ anxiety symptoms with caregivers’ depressive symptoms) pathways in the ITP. In all four APIM models, adolescents’ gender, grade level, only‐child status, caregiver role, parental education level, and annual family income were included as covariates. Model fit was evaluated using the chi‐square statistic (*χ* ^2^), the *p*‐value of *χ* ^2^, the Comparative Fit Index (CFI), the Root Mean Square Error of Approximation (RMSEA), and the Standardized Root Mean Square Residual (SRMR).

Finally, multiple‐group comparison analyses were conducted to examine whether adolescents’ and caregivers’ gender moderated the partner effects in ITP pathways. Taking adolescent gender as an example, we first estimated an unconstrained model in which partner effects were freely estimated across adolescent gender groups (the free model). We then compared it with a constrained model in which partner effects were held equal across adolescent gender groups. The difference in model fit between the two models was evaluated using the Likelihood Ratio Test (LRT) [12]. All statistical analyses were based on the lavaan package in R [29], and a two‐tailed *P* was set to 0.05 as a significant level.

## 4. Results

### 4.1. Sociodemographic Characteristics of Participants

A total of 1414 parent–adolescent dyads completed both waves of the survey. The adolescent sample was nearly evenly split by gender (males: 718, 50.78% and females: 696, 49.22%). Among them, 749 adolescents (52.97%) were primarily cared for by their fathers, while 665 (47.02%) were under maternal care. Most adolescents were enrolled in junior high school (75.67%) and were only children (60.68%). Nearly half of the parents had attained a high school or associate degree (48.23%). In total, 40.88% of families reported a medium household income level.

Group comparisons revealed that junior high school students were more likely to be cared for by their mothers compared to senior high school students (*p* < 0.001). Mothers also reported a significantly higher likelihood of holding a bachelor’s degree or above compared to fathers (*p* < 0.001). In low‐ and medium‐income families, fathers were more likely to be the primary caregivers, whereas in high‐income families, caregiving was more commonly provided by mothers (*p* < 0.001). Detailed descriptive statistics are presented in Table 1.

**Table 1:** Sociodemographic characteristics of participants (*N* = 1414).

Participants characteristics	Total	Father as caregiver (*n* = 749)	Mother as caregiver (*n* = 665)	*χ* ^2^	*P*
Gender (adolescents)					
Male	718 (50.78)	392 (52.34)	326 (49.02)	1.548	0.213
Female	696 (49.22)	357 (47.66)	339 (50.98)	—	—
Grade level (adolescents)					
Junior high school	1,070 (75.67)	470 (62.75)	600 (90.23)	144.442	**<0.001**
Senior high school	344 (24.33)	279 (37.25)	65 (9.77)	—	—
Only‐child (adolescents)					
No	556 (39.32)	293 (39.12)	263 (39.55)	0.027	0.869
Yes	858 (60.68)	456 (60.88)	402 (60.45)	—	—
Education level (caregivers)					
Junior high school or below	292 (20.65)	176 (23.5)	116 (17.44)	37.877	**<0.001**
Senior high school or associate degree	682 (48.23)	393 (52.47)	289 (43.46)	—	—
Bachelor’s degree or above	440 (31.12)	180 (24.03)	260 (39.1)	—	—
Annual income (yuan) of family (caregivers)					
<80,000	342 (24.19)	200 (26.70)	142 (21.35)	30.819	**<0.001**
80,000–150,000	578 (40.88)	337 (44.99)	241 (36.24)	—	—
>150,000	494 (34.94)	212 (28.30)	282 (42.41)	—	—

*Note:* Bold value indicated *p* < 0.05.

### 4.2. Mental Status of Participants

As shown in Table 2, girls reported significantly higher levels of depressive and anxiety symptoms at both T1 and T2 compared to boys (*p* < 0.001). No significant differences were observed in caregivers’ mental health symptoms between adolescents of different genders.

**Table 2:** Depressive and anxiety symptoms of participants and comparison between different groups.

Variable	Total	Boys (50.78%)	Girls (49.22%)	*P*_a_	Fathers (52.97%)	Mothers (47.03%)	*P*_b_	*P*_c_
Adolescents								
T1 Depressive symptoms	3.56 ± 4.58	2.99 ± 4.17	4.14 ± 4.91	**<0.001**	2.95 ± 4.04	4.24 ± 5.04	**<0.001**	**<0.001**
T2 Depressive symptoms	4.09 ± 4.94	3.20 ± 4.08	5.02 ± 5.54	**<0.001**	3.81 ± 4.66	4.41 ± 5.21	**0.049**	—
T1 Anxiety symptoms	3.34 ± 4.19	2.77 ± 3.74	3.93 ± 4.54	**<0.001**	2.74 ± 3.69	4.02 ± 4.60	**<0.001**	**0.015**
T2 Anxiety symptoms	3.67 ± 4.43	2.79 ± 3.82	4.58 ± 4.82	**<0.001**	3.33 ± 3.98	4.06 ± 4.87	0.056	—
Caregivers								
T1 Depressive symptoms	1.85 ± 3.10	1.75 ± 2.94	1.96 ± 3.26	0.105	1.61 ± 2.86	2.12 ± 3.33	**<0.001**	**<0.001**
T2 Depressive symptoms	2.22 ± 3.43	2.34 ± 3.52	2.10 ± 3.32	0.303	1.83 ± 3.20	2.66 ± 3.62	**<0.001**	—
T1 Anxiety symptoms	2.65 ± 3.38	2.49 ± 3.22	2.81 ± 3.54	0.078	2.65 ± 3.33	2.64 ± 3.45	0.858	**<0.001**
T2 Anxiety symptoms	2.09 ± 3.15	2.08 ± 3.20	2.11 ± 3.10	0.770	1.63 ± 2.78	2.62 ± 3.44	**<0.001**	—

*Note: P*_a_: boys vs. girls; *P*_b_: fathers vs. mothers; *P*_c_: T1 vs. T2; Mann–Whitney *U* test for *P*_a_ and *P*_b_ while Wilcoxon signed‐rank test for *P*_c_; Bold value indicated *p* < 0.05; T1 denotes the baseline assessment, and T2 denotes the follow‐up assessment.

Mothers reported higher levels of depressive symptoms at both T1 (*p* < 0.001) and T2 (*p* < 0.001), and significantly higher anxiety symptoms at T2 (*p* < 0.001), compared to fathers. Adolescents cared for by mothers reported significantly higher depressive symptoms at both T1 (*p* < 0.001) and T2 (*p* = 0.049), and significantly higher anxiety symptoms at T1 (*p* < 0.001), compared to those cared for by fathers.

The levels of adolescents’ depressive and anxiety symptoms at T2 were significantly higher than those at T1 (*p* < 0.001 at T1 and *p*  = 0.015 at T2). Caregivers’ depressive symptoms also increased from T1 to T2 (*p* < 0.001). Depressive and anxiety symptoms reported by adolescents at both time points were positively and significantly correlated with caregivers’ depressive and anxiety symptoms at both time points (see Figure S1).

### 4.3. Cross‐Lagged Associations Between Primary Caregivers’ and Adolescents’ Depressive and Anxiety Symptoms

**Figure 1:**
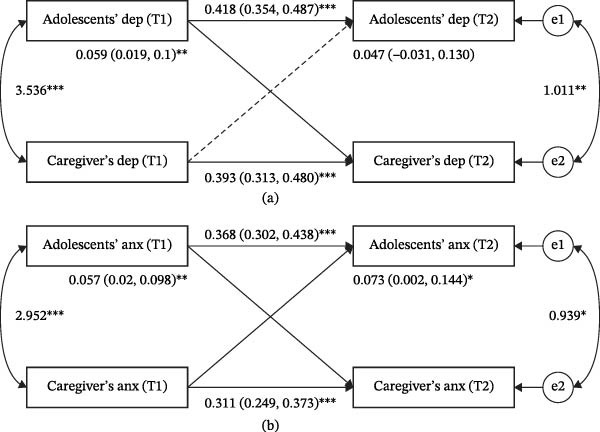
Single‐disorder intergenerational transmission of depressive and anxiety symptoms. (a) Intergenerational transmission of depressive symptoms between adolescents and caregivers. (b) Intergenerational transmission of anxiety symptoms between adolescents and caregivers. *Note:* Dep: depressive symptoms; Anx: anxiety symptoms; the solid line represents significant associations, while the dotted line represents insignificant associations; values are unstandardized betas (95% CI); T1 denotes the baseline assessment, and T2 denotes the follow‐up assessment;  ^∗^ *p* < 0.05,  ^∗∗^ *p* < 0.01,  ^∗∗∗^ *p* < 0.001.

**Figure 2:**
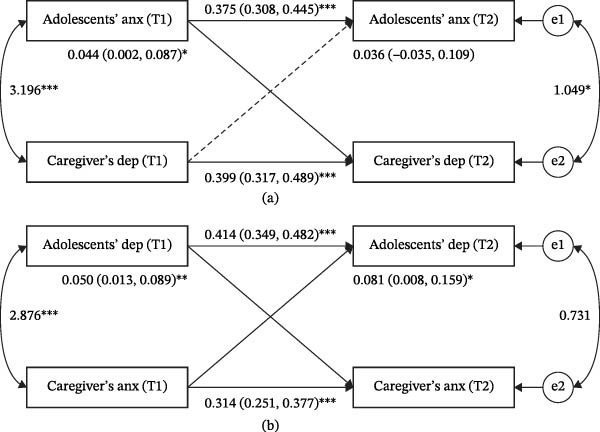
Cross‐disorder intergenerational transmission of depressive and anxiety symptoms. (a) Intergenerational association between adolescents’ anxiety symptoms and caregivers’ depressive symptoms. (b) Intergenerational association between adolescents’ depressive symptoms and caregivers’ anxiety symptoms. *Note:* Dep: depressive symptoms; Anx: anxiety symptoms; the solid line represents significant associations, while the dotted line represents insignificant associations; values are unstandardized betas (95% CI); T1 denotes the baseline assessment, and T2 denotes the follow‐up assessment;  ^∗^ *p* < 0.05,  ^∗∗^ *p* < 0.01,  ^∗∗∗^ *p* < 0.001.

Figures 1 and 2 present the APIM models for single‐disorder transmission and cross‐disorder transmission pathways after adjusting for all covariates, and all models demonstrated good overall fit (Table S1). Significant actor effects were observed for both adolescents and their primary caregivers, indicating that depressive and anxiety symptoms at T1 significantly predicted their own subsequent symptoms at T2.

Regarding partner effects, the results showed that adolescents’ depressive and anxiety symptoms significantly predicted subsequent depressive and anxiety symptoms in their primary caregivers. In contrast, only caregivers’ anxiety symptoms significantly predicted adolescents’ subsequent depressive and anxiety symptoms. No significant associations were found between caregivers’ depressive symptoms and subsequent adolescents’ depressive and anxiety symptoms (Figures 1 and 2).

As is shown in Table 3, all the model fit of the free model was not significantly better than that of the constrained model, indicating that neither adolescent gender nor primary caregiver gender served as a significant moderator of the partner effects (detailed estimates from the free model are listed in Tables S2 and S3).

**Table 3:** The results of multiple groups comparison test.

Models	AIC	BIC	Chisq	Chisq diff	*P*
Caregiver’s gender moderated model					
Caregiver’s Dep–adolescents’ Dep					
Free model	30,846	31,098	55.135	—	—
Constrained model	30,842	31,084	55.871	0.736	0.692
Caregiver’s Anx–adolescents’ Anx					
Free model	30,338	30,590	92.362	—	—
Constrained model	30,335	30,577	93.959	1.598	0.450
Caregiver’s Anx–adolescents’ Dep					
Free model	30,898	31,150	88.562	—	—
Constrained model	30,896	31,138	91.152	2.590	0.274
Caregiver’s Dep–adolescents’ Anx					
Free model	30,310	30,562	55.986	—	—
Constrained model	30,308	30,550	58.357	2.371	0.306
Adolescent’s gender moderated model					
Caregiver’s Dep–adolescents’ Dep					
Free model	30,849	31,101	51.440	—	—
Constrained model	30,847	31,089	53.938	2.498	0.287
Caregiver’s Anx–adolescents’ Anx					
Free model	30,386	30,638	75.501	—	—
Constrained model	30,384	30,625	77.129	1.629	0.443
Caregiver’s Anx–adolescents’ Dep					
Free model	30,913	31,165	72.226	—	—
Constrained model	30,912	31,153	75.032	2.805	0.246
Caregiver’s Dep–adolescents’ Anx					
Free model	30,346	30,598	53.643	—	—
Constrained model	30,343	30,585	54.890	1.247	0.536

*Note:* Dep: depressive symptoms; Anx: anxiety symptoms; T1 denotes the baseline assessment, and T2 denotes the follow‐up assessment.

## 5. Discussion

To the best of our knowledge, this is the first study to investigate the bidirectional transmission of depressive and anxiety symptoms between primary caregivers and adolescents in a Chinese sample. The findings not only confirm the presence of emotional reciprocity within caregiver–adolescent dyads but also reveal that neither adolescent nor caregiver gender significantly moderates the ITP.

The present findings indicated that adolescents’ depressive and anxiety symptoms can significantly predict subsequent depressive and anxiety symptoms in their primary caregivers. Children’s psychopathological symptoms may undermine family cohesion, adaptability, and intrafamily communication, thereby impairing parents’ emotional regulation and contributing to their psychological distress [7, 30].

However, our study further revealed that only caregivers’ anxiety symptoms, not their depressive symptoms, significantly predicted adolescents’ subsequent depressive and anxiety symptoms. One possible explanation is that caregiver anxiety may be more salient and readily perceived by adolescents. Prior research has shown that caregiver anxiety influences children’s socio‐emotional development and psychopathological outcomes through overinvolved critical parenting behaviors and psychological control [4, 31–33]. Compared to depressive symptoms, which are often characterized by low energy and withdrawal, anxiety symptoms may be more externally expressed and observable [34]. Anxious parents tend to exhibit more pronounced behavioral and verbal stress cues, which can increase children’s awareness of stress and threat, ultimately affecting their emotional regulation and mental health [4, 35]. Additionally, this pattern may be influenced by the specific sociocultural context of the study. In traditional Chinese Confucian culture, the overt expression of negative emotions by adults is a sign of weakness [36]. Parents tend to suppress emotional expression and adopt the role of a strong, stoic caregiver figure [37, 38]. Consequently, parents may suppress emotional expression and instead adopt a stoic and strong parental role [37, 38], which could attenuate the top‐down transmission of depressive symptoms [39].

Contrary to our hypothesis, we did not find any significant moderating effects of the caregiver or adolescent gender in the ITP. Traditionally, it has been assumed that mothers are more involved in monitoring and caregiving responsibilities than fathers [40]. However, our findings showed that adolescents were nearly equally likely to be primarily cared for by their fathers or mothers, suggesting that fathers are now more actively involved in adolescent development, and the strict division of caregiving roles suggested by traditional stereotypes may no longer hold [41]. In addition, societal and cultural changes in China have contributed to a shift in parental roles. Research has shown a decline in the traditional “strict father, kind mother” parenting pattern and the emergence of a more contemporary model characterized by “tiger moms and panda dads,” reflecting the evolving roles of mothers and fathers in adolescent development [41]. Although our univariate analyses indicated that adolescents cared for by mothers reported higher levels of depressive and anxiety symptoms compared to those cared for by fathers, these results were not adjusted for potential confounding variables and should therefore be interpreted with caution.

Finally, contrary to the gender intensification hypothesis [16], the present study did not find evidence that adolescent gender moderates ITP. In fact, the gender intensification hypothesis has been challenged by prior research [19]. A meta‐analysis by Yap et al. [42] reviewed 68 studies and found that only 6 reported significant moderating effects of adolescent gender on the association between parental factors and youth psychological symptoms—and all six were conducted in clinical samples of adolescents with diagnosed depression. However, Hastings et al. [12] also found that maternal depression predicted depressive symptoms specifically in daughters within community‐based families in the United States. Further research is needed to determine whether these divergent findings are driven by specific population characteristics or sociocultural contexts.

These findings carry important implications for interventions targeting ITP. First, beyond the conventional three‐tiered prevention strategies for adolescent depression and anxiety, greater attention should be paid to the emotional well‐being of caregivers. Psychoeducation for parents and family‐based interventions addressing caregivers’ mental health may help disrupt the ITP pathway. Second, while many existing interventions primarily focus on parental depression, our findings suggest that caregiver anxiety may serve as a critical and potentially modifiable target in the transmission process. Expanding current intervention frameworks to include anxiety management strategies for caregivers may enhance their effectiveness in preventing the intergenerational perpetuation of emotional distress.

There are several limitations in this study. First, the representativeness of the sample may be limited by its single‐center design and cultural context. Second, the two‐wave investigation with only a 1‐year interval may not capture long‐term changes in ITP. Third, the prospective associations identified here, without an experimental or genetically informed design, cannot be interpreted as causal. Fourth, due to constraints in survey timing and item sensitivity, we included only a limited set of covariates and were unable to account for genetic information such as the family history of psychiatric disorders. Fifth, this study focused on transactional patterns of depressive and anxiety symptoms between primary caregivers and adolescents but did not assess the influence of the other parental partner, which may have confounded the results.

## 6. Conclusions

Caregivers’ anxiety symptoms significantly predicted subsequent depressive and anxiety symptoms in adolescents, while adolescents’ depressive and anxiety symptoms predicted subsequent symptoms in their caregivers. No moderating effects of caregiver or adolescent gender were observed in the ITP. These findings highlight adolescents’ depressive and anxiety symptoms and caregivers’ anxiety symptoms as key intervention targets for preventing ITP. A family‐based approach to early identification and prevention could be a promising direction for interrupting the escalating cycles of negative emotions across generations.

## Author Contributions

Conceptualization, data curation, investigation: Hao Hou and Dan Luo. Formal analysis, software, methodology, visualization: Hao Hou. Project administration: Dan Luo, Huijing Zou, and Bing Xiang Yang. Funding acquisition: Dan Luo and Bing Xiang Yang. Supervision: Dan Luo and Bing Xiang Yang. Writing – original draft preparation: Hao Hou, Dan Luo, Shuzhen Zhu, Si Chen Zhou, Shu Yan, Yu Lei Jiang, Xiao Qin Wang, Qian Liu, Huijing Zou, and Bing Xiang Yang.

## Funding

This work was supported by grants from the National Natural Science Foundation of China (Grants 72474166, 72304212, and 72174152), the National Key Research and Development Project of China (Grant 2024YFC3308400), the Young Top‐notch Talent Cultivation Program of Hubei Province, and the Students’ Mental Health Network Project.

## Disclosure

All authors have reviewed and approved the submitted version of the manuscript and have agreed to be accountable for all aspects of the work.

## Ethics Statement

The protocol for this research was approved by the Clinical Research Ethics Committee of the Wuhan Mental Health Center (KY2021.11.01). The procedures used in this study adhere to the tenets of the Declaration of Helsinki. All participants completed written informed consent before inclusion in the study.

## Conflicts of Interest

The authors declare no conflicts of interest.

## Supporting Information

Additional supporting information can be found online in the Supporting Information section.

## Supporting information


**Supporting Information** Figure S1 demonstrated the Spearman correlations of adolescents’ and primary caregivers’ depressive and anxiety symptoms. Table S1 demonstrated the model fit of the main‐effect model (controlled for main caregivers’ and adolescents’ gender and other covariates). Table S2 showed the detailed model coefficients of the main‐effect model, stratified by the primary caregiver’s gender (while controlling for adolescents’ gender and other covariates). Table S3 showed the detailed model coefficients of the main‐effect model, stratified by adolescents’ gender (while controlling for primary caregiver’s gender and other covariates).

## Data Availability

The data that support the findings of this study are available from the corresponding author upon reasonable request.
