# Supplementary material for: Bidirectional Associations Between Primary Caregivers’ and Adolescents’ Depressive and Anxiety Symptoms: An Intergenerational Actor–Partner Interdependence Model Analysis
Source: Depress Anxiety. 2026 May 30;2026:6987736. doi: 10.1155/da/6987736 (PMC13239181; doi:10.1155/da/6987736)
Supplement: Supplementary file 1 — Supporting Information Figure S1 demonstrated the Spearman correlations of adolescents’ and primary caregivers’ depressive and anxiety symptoms. Table S1 demonstrated the model fit of the main‐effect model (controlled for main caregivers’ and adolescents’ gender and other covariates). Table S2 showed the detailed model coefficients of the main‐effect model, stratified by the primary caregiver’s gender (while controlling for adolescents’ gender and other covariates). Table S3 showed the detailed model coefficients of the main‐effect model, stratified by adolescents’ gender (while controlling for primary caregiver’s gender and other covariates). [file DA-2026-6987736-s001.docx]

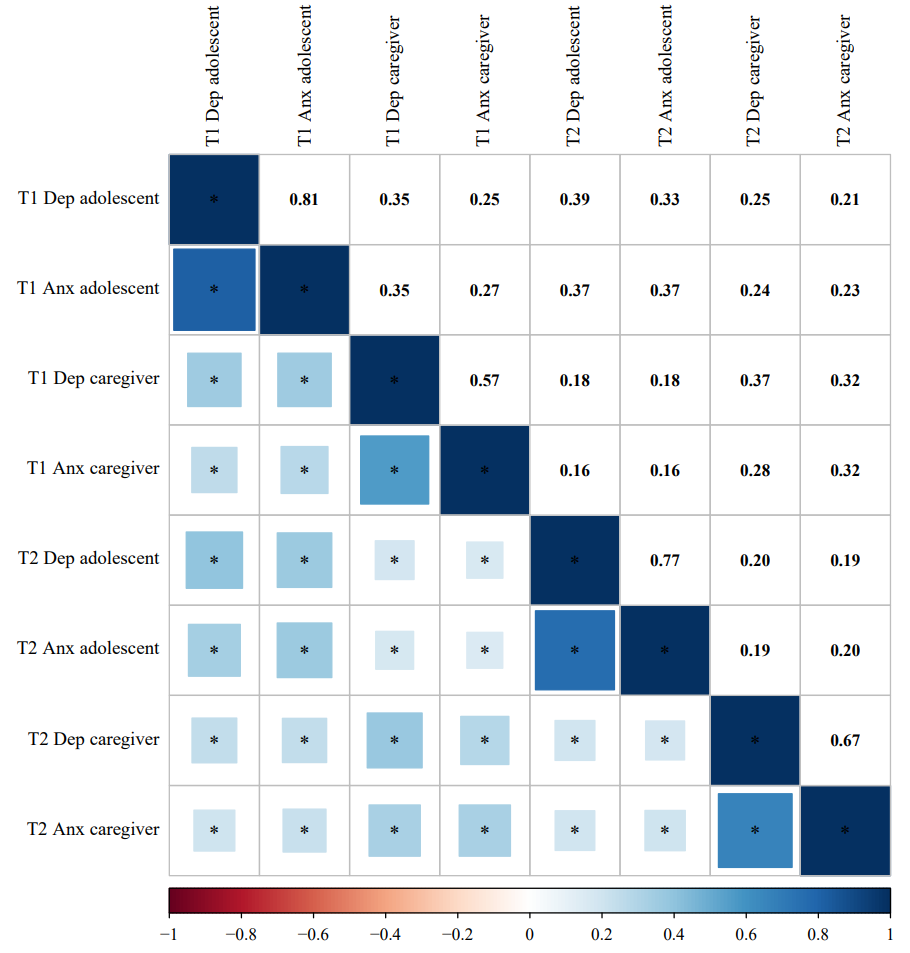


**Figure S1.** Spearman correlations of adolescents’ and primary caregivers’ depressive and anxiety symptoms at baseline (T1) and follow up (T2)

Note: * <0.05; Dep: depressive symptoms; Anx: anxiety symptoms

**Table S1.** Cross-lagged associations between primary caregivers’ and adolescents’ depressive and anxiety symptoms (main effect model).

| Models | Role | Effect | Predictor | Outcome | Estimate (95%CI) | P | Model fit | | | | |
| --- | --- | --- | --- | --- | --- | --- | --- | --- | --- | --- | --- |
|  |  |  |  |  |  |  | χ² | p of χ² | CFI | RMSEA | SRMR |
| Main effect model (Caregiver's Dep-Adolescents' Dep) | | | | | | | | | | | |
|  | Adolescents | Actor effect | T1AD | T2AD | 0.418 (0.354,0.487) | <0.001 | 62.169 | <0.001 | 0.928 | 0.054 | 0.033 |
|  |  | Partner effect | T1AD | T2PD | 0.059 (0.019,0.100) | 0.005 |  |  |  |  |  |
|  | Caregivers | Actor effect | T1PD | T2PD | 0.393 (0.313,0.480) | <0.001 |  |  |  |  |  |
|  |  | Partner effect | T1PD | T2AD | 0.047 (-0.031,0.130) | 0.249 |  |  |  |  |  |
| Main effect model (Caregiver's Anx-Adolescents' Anx) | | | | | | | | | | | |
|  | Adolescents | Actor effect | T1AA | T2AA | 0.368 (0.302,0.438) | <0.001 | 93.503 | <0.001 | 0.878 | 0.069 | 0.036 |
|  |  | Partner effect | T1AA | T2PA | 0.057 (0.020,0.098) | 0.004 |  |  |  |  |  |
|  | Caregivers | Actor effect | T1PA | T2PA | 0.311 (0.249,0.373) | <0.001 |  |  |  |  |  |
|  |  | Partner effect | T1PA | T2AA | 0.073 (0.002,0.144) | 0.043 |  |  |  |  |  |
| Main effect model (Caregiver's Anx-Adolescents' Dep) | | | | | | | | | | | |
|  | Adolescents | Actor effect | T1AD | T2AD | 0.414 (0.349,0.482) | <0.001 | 83.016 | <0.001 | 0.894 | 0.065 | 0.034 |
|  |  | Partner effect | T1AD | T2PA | 0.050 (0.013,0.089) | 0.010 |  |  |  |  |  |
|  | Caregivers | Actor effect | T1PA | T2PA | 0.314 (0.251,0.377) | <0.001 |  |  |  |  |  |
|  |  | Partner effect | T1PA | T2AD | 0.081 (0.008,0.159) | 0.038 |  |  |  |  |  |
| Main effect model (Caregiver's Dep-Adolescents' Anx) | | | | | | | | | | | |
|  | Adolescents | Actor effect | T1AA | T2AA | 0.375 (0.308,0.445) | <0.001 | 71.418 | <0.001 | 0.912 | 0.059 | 0.035 |
|  |  | Partner effect | T1AA | T2PD | 0.044 (0.002,0.087) | 0.040 |  |  |  |  |  |
|  | Caregivers | Actor effect | T1PD | T2PD | 0.399 (0.317,0.489) | <0.001 |  |  |  |  |  |
|  |  | Partner effect | T1PD | T2AA | 0.036 (-0.035,0.109) | 0.324 |  |  |  |  |  |

Note: AD: adolescents’ depressive symptoms; AA: adolescents’ anxiety symptoms; PD: caregivers’ depressive symptoms; PA: caregivers’ anxiety symptoms; Estimate (95%CI): unstandardized betas (95%CI).

**Table S2.** Cross-lagged associations between primary caregivers’ and adolescents’ depressive and anxiety symptoms by caregiver gender (free model results)

| Free models | Moderated model | Role | Effect | Predictor | Outcome | Estimate (95%CI) | P |  |
| --- | --- | --- | --- | --- | --- | --- | --- | --- |
|  |  |  |  |  |  |  |  |  |
| Caregiver's Dep-Adolescents' Dep | | | |  |  |  |  |  |
|  | Father as main caregiver | Adolescents | Actor effect | T1AD | T2AD | 0.424 (0.330,0.522) | <0.001 |  |
|  |  |  | Partner effect | T1AD | T2PD | 0.047 (-0.016,0.113) | 0.152 |  |
|  |  | Fathers | Actor effect | T1PD | T2PD | 0.326 (0.203,0.459) | <0.001 |  |
|  |  |  | Partner effect | T1PD | T2AD | 0.017 (-0.099,0.131) | 0.764 |  |
|  | Mother as main caregiver | Adolescents | Actor effect | T1AD | T2AD | 0.412 (0.321,0.506) | <0.001 |  |
|  |  |  | Partner effect | T1AD | T2PD | 0.067 (0.015,0.119) | 0.011 |  |
|  |  | Mothers | Actor effect | T1PD | T2PD | 0.446 (0.341,0.561) | <0.001 |  |
|  |  |  | Partner effect | T1PD | T2AD | 0.071 (-0.04,0.194) | 0.225 |  |
| Caregiver's Anx-Adolescents' Anx | | | |  |  |  |  |  |
|  | Father as main caregiver | Adolescents | Actor effect | T1AA | T2AA | 0.424 (0.330,0.522) | <0.001 |  |
|  |  |  | Partner effect | T1AA | T2PA | 0.047 (-0.016,0.113) | 0.013 |  |
|  |  | Fathers | Actor effect | T1PA | T2PA | 0.326 (0.203,0.459) | <0.001 |  |
|  |  |  | Partner effect | T1PA | T2AA | 0.017 (-0.099,0.131) | 0.429 |  |
|  | Mother as main caregiver | Adolescents | Actor effect | T1AA | T2AA | 0.412 (0.321,0.506) | <0.001 |  |
|  |  |  | Partner effect | T1AA | T2PA | 0.067 (0.015,0.119) | 0.164 |  |
|  |  | Mothers | Actor effect | T1PA | T2PA | 0.446 (0.341,0.561) | <0.001 |  |
|  |  |  | Partner effect | T1PA | T2AA | 0.071 (-0.040,0.194) | 0.086 |  |
| Caregiver's Anx-Adolescents' Dep | | | |  |  |  |  |  |
|  | Father as main caregiver | Adolescents | Actor effect | T1AD | T2AD | 0.425 (0.331,0.516) | <0.001 |  |
|  |  |  | Partner effect | T1AD | T2PA | 0.062 (0.014,0.114) | 0.015 |  |
|  |  | Caregivers | Actor effect | T1PA | T2PA | 0.172 (0.103,0.250) | <0.001 |  |
|  |  |  | Partner effect | T1PA | T2AD | 0.023 (-0.080,0.131) | 0.664 |  |
|  | Mother as main caregiver | Adolescents | Actor effect | T1AD | T2AD | 0.403 (0.312,0.497) | <0.001 |  |
|  |  |  | Partner effect | T1AD | T2PA | 0.031 (-0.024,0.088) | 0.269 |  |
|  |  | Caregivers | Actor effect | T1PA | T2PA | 0.466 (0.373,0.561) | <0.001 |  |
|  |  |  | Partner effect | T1PA | T2AD | 0.121 (0.008,0.243) | 0.041 |  |
| Caregiver's Dep-Adolescents' Anx | | | |  |  |  |  |  |
|  | Father as main caregiver | Adolescents | Actor effect | T1AA | T2AA | 0.292 (0.195,0.387) | <0.001 |  |
|  |  |  | Partner effect | T1AA | T2PD | 0.005 (-0.061,0.075) | 0.880 |  |
|  |  | Caregivers | Actor effect | T1PD | T2PD | 0.345 (0.215,0.482) | <0.001 |  |
|  |  |  | Partner effect | T1PD | T2AA | 0.039 (-0.062,0.143) | 0.454 |  |
|  | Mother as main caregiver | Adolescents | Actor effect | T1AA | T2AA | 0.427 (0.335,0.526) | <0.001 |  |
|  |  |  | Partner effect | T1AA | T2PD | 0.071 (0.018,0.128) | 0.012 |  |
|  |  | Caregivers | Actor effect | T1PD | T2PD | 0.447 (0.341,0.564) | <0.001 |  |
|  |  |  | Partner effect | T1PD | T2AA | 0.042 (-0.057,0.149) | 0.421 |  |

Note: AD: adolescents’ depressive symptoms; AA: adolescents’ anxiety symptoms; PD: caregivers’ depressive symptoms; PA: caregivers’ anxiety symptoms; Estimate (95%CI): unstandardized betas (95%CI).

**Table S3.** Cross-lagged associations between primary caregivers’ and adolescents’ depressive and anxiety symptoms by adolescent gender (free model results)

| Free models | Moderated model | Role | Effect | Predictor | Outcome | Estimate (95%CI) | P |  |
| --- | --- | --- | --- | --- | --- | --- | --- | --- |
|  |  |  |  |  |  |  |  |  |
| Caregiver's Dep-Adolescents' Dep | | | | | | | |  |
|  | Male adolescents | Boys | Actor effect | T1AD | T2AD | 0.328 (0.242,0.414) | <0.001 |  |
|  |  |  | Partner effect | T1AD | T2PD | 0.088 (0.026,0.155) | 0.007 |  |
|  |  | Caregivers | Actor effect | T1PD | T2PD | 0.434 (0.314,0.559) | <0.001 |  |
|  |  |  | Partner effect | T1PD | T2AD | 0.086 (-0.027,0.208) | 0.144 |  |
|  | Female adolescents | Girls | Actor effect | T1AD | T2AD | 0.484 (0.387,0.580) | <0.001 |  |
|  |  |  | Partner effect | T1AD | T2PD | 0.038 (-0.014,0.09) | 0.154 |  |
|  |  | Caregivers | Actor effect | T1PD | T2PD | 0.358 (0.254,0.477) | <0.001 |  |
|  |  |  | Partner effect | T1PD | T2AD | 0.018 (-0.092,0.137) | 0.754 |  |
| Caregiver's Anx-Adolescents' Anx | | | | | | | |  |
|  | Male adolescents | Boys | Actor effect | T1AA | T2AA | 0.334 (0.237,0.440) | <0.001 |  |
|  |  |  | Partner effect | T1AA | T2PA | 0.086 (0.026,0.153) | 0.008 |  |
|  |  | Caregivers | Actor effect | T1PA | T2PA | 0.337 (0.237,0.440) | <0.001 |  |
|  |  |  | Partner effect | T1PA | T2AA | 0.083 (-0.015,0.181) | 0.103 |  |
|  | Female adolescents | Girls | Actor effect | T1AA | T2AA | 0.392 (0.302,0.480) | <0.001 |  |
|  |  |  | Partner effect | T1AA | T2PA | 0.038 (-0.01,0.091) | 0.144 |  |
|  |  | Caregivers | Actor effect | T1PA | T2PA | 0.290 (0.213,0.368) | <0.001 |  |
|  |  |  | Partner effect | T1PA | T2AA | 0.067 (-0.037,0.172) | 0.210 |  |
| Caregiver's Anx-Adolescents' Dep | | | | | | | |  |
|  | Male adolescents | Boys | Actor effect | T1AD | T2AD | 0.322 (0.239,0.408) | <0.001 |  |
|  |  |  | Partner effect | T1AD | T2PA | 0.072 (0.014,0.133) | 0.019 |  |
|  |  | Caregivers | Actor effect | T1PA | T2PA | 0.339 (0.236,0.445) | <0.001 |  |
|  |  |  | Partner effect | T1PA | T2AD | 0.133 (0.027,0.240) | 0.014 |  |
|  | Female adolescents | Girls | Actor effect | T1AD | T2AD | 0.481 (0.388,0.575) | <0.001 |  |
|  |  |  | Partner effect | T1AD | T2PA | 0.033 (-0.015,0.084) | 0.189 |  |
|  |  | Caregivers | Actor effect | T1PA | T2PA | 0.292 (0.217,0.370) | <0.001 |  |
|  |  |  | Partner effect | T1PA | T2AD | 0.045 (-0.065,0.161) | 0.440 |  |
| Caregiver's Dep-Adolescents' Anx | | | | | | | |  |
|  | Male adolescents | Boys | Actor effect | T1AA | T2AA | 0.339 (0.238,0.449) | <0.001 |  |
|  |  |  | Partner effect | T1AA | T2PD | 0.070 (0.003,0.143) | 0.049 |  |
|  |  | Caregivers | Actor effect | T1PD | T2PD | 0.446 (0.324,0.574) | <0.001 |  |
|  |  |  | Partner effect | T1PD | T2AA | 0.056 (-0.05,0.169) | 0.320 |  |
|  | Female adolescents | Girls | Actor effect | T1AA | T2AA | 0.400 (0.310,0.490) | <0.001 |  |
|  |  |  | Partner effect | T1AA | T2PD | 0.028 (-0.025,0.083) | 0.304 |  |
|  |  | Caregivers | Actor effect | T1PD | T2PD | 0.361 (0.249,0.485) | <0.001 |  |
|  |  |  | Partner effect | T1PD | T2AA | 0.019 (-0.078,0.123) | 0.710 |  |

Note: AD: adolescents’ depressive symptoms; AA: adolescents’ anxiety symptoms; PD: caregivers’ depressive symptoms; PA: caregivers’ anxiety symptoms; Estimate (95%CI): unstandardized betas (95%CI).
